# Current Trends and Perspectives of Pressure Wire-Based Coronary Artery Bypass Grafting

**DOI:** 10.3390/jcdd12010016

**Published:** 2025-01-02

**Authors:** Yoshiyuki Takami, Atsuo Maekawa, Koji Yamana, Kiyotoshi Akita, Kentaro Amano, Wakana Niwa, Kazuki Matsuhashi, Yasushi Takagi

**Affiliations:** Department of Cardiovascular Surgery, Fujita Health University School of Medicine, 1-98 Dengakugakubo, Kutsukake, Toyoake 470-1192, Aichi, Japan

**Keywords:** coronary artery bypass grafting, fractional flow reserve, non-hyperemic pressure ratios, coronary computed tomography angiography

## Abstract

Fractional flow reserve (FFR) has been well validated as a modality for evaluating myocardial ischemia, demonstrating the superiority of FFR-guided percutaneous coronary intervention (PCI) over conventional angiography-guided PCI. As a result, the strategy for coronary artery bypass grafting (CABG) is shifting toward FFR guidance. However, the advantage of FFR-guided CABG over angiography-guided CABG remains unclear. While FFR-guided CABG can help avoid unnecessary grafting in cases of moderate stenosis, it may also carry the risk of incomplete revascularization. The limited use of FFR due to the need for hyperemia has led to the development of non-hyperemic pressure ratios (NHPRs). NHPR pullback provides trans-stenotic pressure gradients, which may offer valuable insights for CABG strategies. Recently, computed tomographic coronary angiography (CTCA) has emerged as a non-invasive modality that provides accurate data on lesion length, diameter, minimum lumen area, percentage stenosis, and the volume and distribution of high-risk plaques. With the introduction of FFR-CT, CTCA is now highly anticipated to provide both functional evaluation (of myocardial ischemia) via FFR-CT and anatomical information through serial quantitative assessment. Beyond the diagnostic phase, CTCA, augmented by automatic artificial intelligence, holds great potential for guiding therapeutic interventions in the future.

## 1. Introduction

Invasive coronary angiography remains the gold standard for imaging the luminal structures of epicardial coronary arteries and for diagnosing and evaluating coronary artery disease (CAD) [1]. However, it is insufficient for evaluating myocardial ischemia and plaque characteristics, both of which play a critical role in the prognosis of patients with CAD [2]. To assess ischemia, various non-invasive functional imaging modalities have been developed, including dobutamine stress echocardiography, single-photon emission computed tomography (SPECT), stress myocardial perfusion imaging (SPECT-MPI), and cardiac magnetic resonance (MRI) stress imaging and perfusion techniques [3].

In addition to these, fractional flow reserve (FFR)—the ratio of distal coronary pressure to proximal pressure measured under the conditions of maximal hyperemia—has been well validated as a determinant of lesion-specific myocardial ischemia in epicardial CAD [4,5]. Many clinical studies have established an FFR cutoff range of 0.75 to 0.80 to distinguish between nonfunctionally significant and functionally significant coronary artery stenosis [6]. The FFR Versus Angiography for Multivessel Evaluation (FAME) study demonstrated the superiority of FFR-guided percutaneous coronary intervention (PCI) over conventional angiography-guided PCI [5]. Subsequently, the FAME-2 trial showed that FFR-guided PCI outperformed medical therapy alone [7], primarily by safely deferring lesions that appeared stenotic but did not cause lesion-specific ischemia, as indicated by FFR. As a result, current guidelines recommend using FFR to complement coronary angiography [8,9].

Under these circumstances, the strategy for coronary artery bypass grafting (CABG) is also shifting toward FFR-guided approaches, although its advantage over angiography-guided CABG remains unclear [6]. Additionally, new physiological indices have been developed with the aim of reducing the procedural and invasive aspects of FFR assessment. These include non-hyperemic pressure ratios (NHPRs), such as the instantaneous wave-free ratio (iFR), diastolic pressure ratio (dPR), diastolic hyperemia-free ratio (DFR), and resting full-cycle ratio (RFR) [10]. This review article describes the current status of pressure wire-based CABG using FFR and NHPRs. Furthermore, we discuss the future perspectives of pressure wire-based CABG, beyond the anatomical and functional evaluation of CAD, for surgical coronary revascularization.

## 2. FFR-Guided CABG

### 2.1. Previous Studies

The use of preoperative FFR for coronary lesions to determine CABG strategy has increasingly garnered attention, despite a lack of evidence showing an improvement in clinical outcomes [6,11,12,13,14,15,16,17]. The earliest study on FFR-guided CABG by Botman et al. revealed a strong correlation between FFR values and patency rates, with FFR > 0.75 resulting in a graft occlusion rate of 21.4% [11]. A second retrospective study by Toth et al. reported that FFR-guided CABG was associated with a significantly lower incidence of angina compared to angiography-guided CABG, despite no difference in major adverse cardiovascular events at the 3-year follow-up [12]. The first prospective study, the FARGO trial, showed similar graft failure rates in FFR-guided and angiography-guided patients [13]. A second prospective study, the 2019 GRAFFITI trial, concluded that the FFR-guided group, which had fewer anastomoses per patient, did not show any impact on one-year graft patency [14]. The third prospective study, the IMPAG trial of FFR-guided multi-arterial CABG, reported that an FFR ≤ 0.78 was associated with a 6-month anastomotic occlusion rate of only 3% [15]. Fournier et al. reported 6-year outcomes showing that FFR-guided CABG reduced overall mortality compared with angiography-guided CABG [16]. Based on these results, a recent review proposed multi-arterial CABG for lesions with FFR ≤ 0.78, and CABG with left internal thoracic artery (LITA) and saphenous vein grafts (SVGs) for lesions with FFR between 0.78 and 0.80 [6].

However, the relative benefits of CABG compared to PCI may be diminished due to a shift toward FFR-guided CABG, which reduces the number of grafted vessels and increases the rate of anatomically defined incomplete revascularization.

### 2.2. Our Current Practice

The idea of making strict revascularization decisions based on a fixed FFR cutoff has gained acceptance, leading to the incorporation of FFR into clinical guidelines, which currently recommend its use based on a fixed cutoff of 0.8 [1]. The DEFER (Deferral Versus Performance of PTCA in Patients Without Documented Ischemia) trial [18] demonstrated the safety of a deferral strategy for stenoses with an FFR ≥ 0.75, whereas the FAME [5] and FAME 2 [7] studies used an FFR cutoff of 0.80 to investigate its value in guiding angioplasty. Therefore, we refer to the range between 0.75 and 0.80 as the DEFER-FAME gray zone. An FFR < 0.75 has 100% specificity for identifying stenoses with inducible ischemia, whereas an FFR > 0.80 has a sensitivity of more than 90% for excluding stenoses that cause myocardial ischemia.

For FFR-guided CABG, a recent review by Spadaccio et al. recommended multivessel CABG with arterial grafts when FFR < 0.78, and CABG using the internal thoracic artery (ITA) and saphenous vein grafts (SVGs) when FFR is between 0.78 and 0.80 [6].

**Figure 1:**
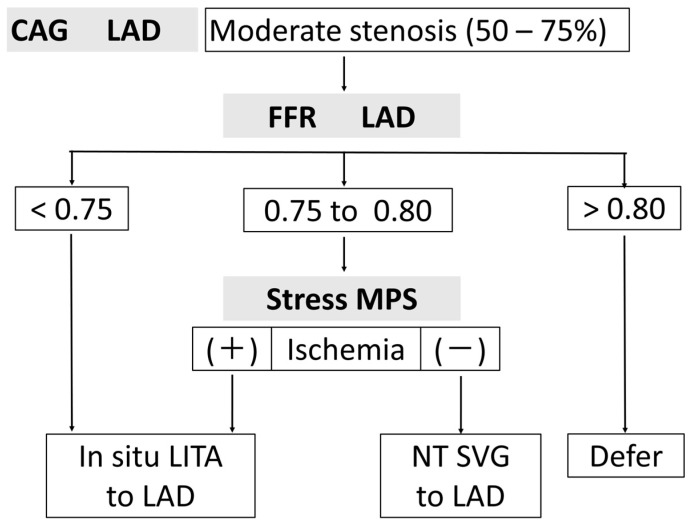
Our current algorithm of FFR-guided revascularization for LAD with moderate stenosis. CAG: coronary angiography, LAD: left anterior descending artery, FFR: fractional flow reserve, MPS: myocardial perfusion scintigraphy, LITA: left internal thoracic artery, NT SVG: no-touch saphenous vein graft.

Based on these considerations, we apply FFR-guided revascularization for the left anterior descending artery (LAD), the most important coronary artery, using our current algorithm (Figure 1). When FFR is < 0.75, in situ left internal thoracic artery (LITA) grafting is performed for the LAD. When FFR is > 0.80, grafting is deferred. In the gray zone (FFR between 0.75 and 0.80), stress myocardial perfusion scintigraphy is performed. A ischemia-positive result in the LAD area leads to in situ LITA grafting to the LAD, while a ischemia-negative result leads to aortocoronary bypass using a no-touch SVG to the LAD. This approach takes into account the string phenomenon of the LITA graft to the LAD with mild stenosis [19] and the higher patency rate of no-touch SVGs [20,21].

**Figure 2:**
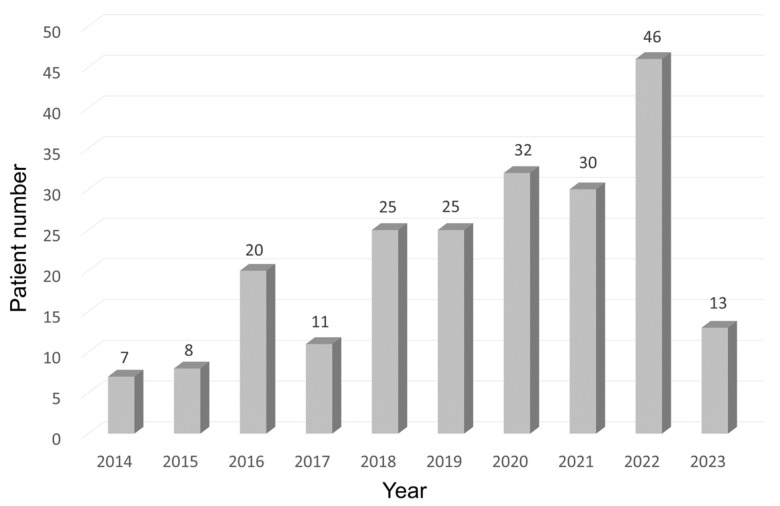
Annual trends in the number of patients who underwent FFR measurement before open-heart surgery at our institution. FFR: fractional flow reserve.

By the end of May 2023, FFR was measured on 266 vessels in 217 patients prior to open-heart surgery, accounting for 15.2% of patients undergoing CABG. The number of patients who have had their FFR measured has been increasing year by year (Figure 2). As shown in Table 1, among the 266 vessels, 140 (53%) were treated with CABG, while 126 (47%) were deferred. The treated coronary lesions included 98 LAD (70%), 2 diagonal branches (1%), 15 left circumflex arteries (LCX) (11%), and 25 right coronary arteries (RCA) (18%). The number of treated LADs was significantly higher than the number of deferred LADs. The treated coronary arteries, including 74 LITA to LAD, 24 SVG to LAD, 15 SVG to LCX, and 24 SVG to RCA, were primarily revascularized during CABG and valve surgery, while the deferred vessels were mostly examined before valve and aortic surgery.

### 2.3. Relation to Graft Flow

As the current European guidelines on myocardial revascularization recommend [17,22], transit-time flow measurement (TTFM) (Medi-Stim, Oslo, Norway) is increasingly used for intraoperative graft flow analysis during CABG as a less invasive, more reproducible, and less time-consuming method. TTFM is based on the principle of calculating blood flow volume by measuring the difference in arrival time between ultrasonic transmissions from the upstream and downstream sides. In addition to morphological assessment using color Doppler, mean graft flow (Qm) > 15 mL/min, pulsatility index (PI) < 5.0, diastolic filling (DF) > 50%, and systolic reverse flow (SRF) < 4% are indicative TTFM variables of a patent graft during CABG [23,24].

Honda et al. [25] reported significantly lower Qm and higher PI in LITA grafts to the left anterior descending artery (LAD) with FFR ≥ 0.75 compared to those with FFR < 0.75. We also reported that most TTFM variables of the LITA-LAD graft are strongly influenced by FFRLAD [26]. Additionally, we found similar correlations between FFRLAD values and Qmin, Qm, PI, and SRF in the SVG to LAD [27]. Therefore, the FFR value of the target coronary artery strongly affects the TTFM variables of the graft. However, Di Giammarco et al. showed that Qm < 15 mL/min, PI > 3, and SRF < 3% predict graft failure after one year [28]. Similarly, Tokuda et al. reported that Qm, PI, and SRF predict mid-term (1 to 4 years) graft failure [29]. Lehnert et al. quantitatively demonstrated that a decrease in Qm by 1 mL/min increased graft failure rates by 4% for LITA and 2% for SVG [30]. Recently, Kim et al. reported that it is Qm and PI, not anastomotic types or targets, that affect long-term graft patency rates [31]. Therefore, we can conclude that higher FFR values without evident myocardial ischemia may lead to worse TTFM indices, which could result in decreased graft patency.

On the other hand, there has been ongoing debate, even before the advent of FFR, regarding whether moderately stenotic lesions should be grafted or deferred. Hayward et al. demonstrated that grafted vessels had a greater risk of disease progression than ungrafted equivalents in all territories, especially in the right coronary territory [32]. In 2018, the Cleveland Clinic reported a study involving more than 1000 patients, showing that disease progression of moderately stenotic lesions occurred faster in grafted vessels than in ungrafted vessels, with a greater effect seen in SVGs compared to ITA grafts [33].

**Figure 3:**
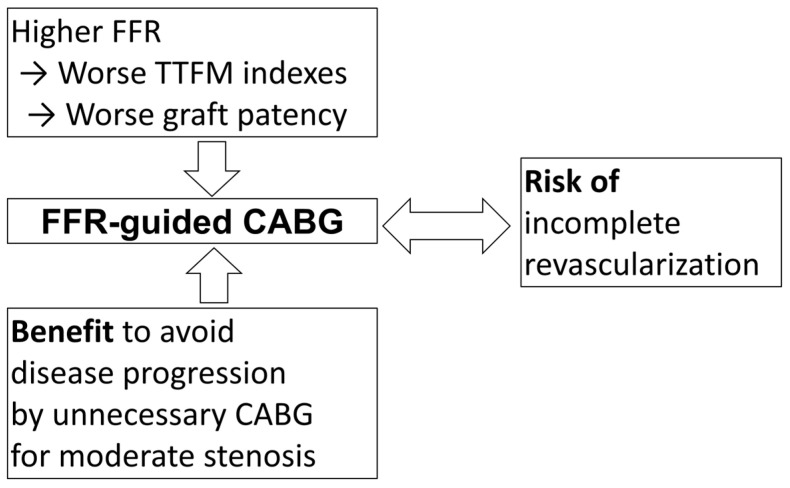
Influencing factors on application of FFR-guided CABG. FFR: fractional flow reserve, CABG: coronary artery bypass grafting.

In summary, we should consider the following points for FFR-guided CABG (Figure 3): (i) a higher FFR value may lead to worse TTFM indices, which could result in decreased graft patency, (ii) FFR-guided CABG may help avoid the risk of disease progression by preventing unnecessary grafting for moderate stenosis, and (iii) FFR-guided CABG may carry a risk of incomplete revascularization.

## 3. NHPRs

### 3.1. Definitions

The application of FFR in daily practice is limited by various factors, including invasive instrumentation of the coronary artery, which requires additional time, cost, and carries the risk of side effects from vasodilator medications used for hyperemia [34]. New physiological indices, such as NHPRs, have been developed to reduce the procedural and invasive aspects of FFR measurement, including iFR, dPR, DFR, and RFR [10,34]. The iFR is a wire-based NHPR that evaluates Pd/Pa during a specific phase of diastole, known as the wave-free period, during which Pd/Pa represents the ratio of mean distal coronary pressure (Pd) to mean aortic pressure (Pa). The dPR is a resting ratio of the mean diastolic pressure distal to the stenosis to the mean diastolic aortic pressure. The DFR is a resting index derived from the average Pd/Pa during the period when the Pa is less than the mean Pa and exhibits a downward slope. The RFR represents the lowest instantaneous Pd/Pa ratio within the entire cardiac cycle. The cutoff value of NHPRs for predicting FFR < 0.80, which indicates myocardial ischemia, is 0.89.

### 3.2. Our Practice

**Figure 4:**
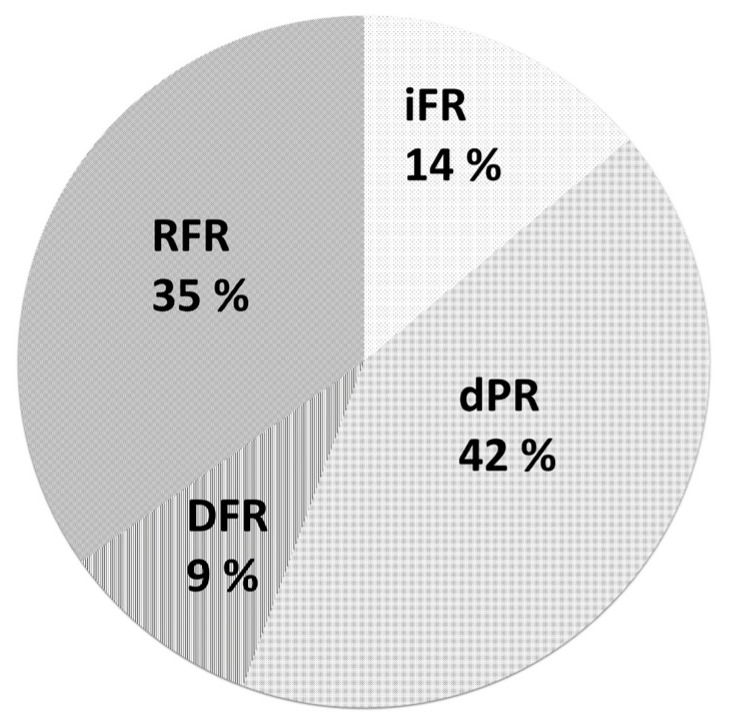
NHPRs measured simultaneously with FFR before open-heart surgery by the end of May 2023 at our institute. NHPRs: non-hyperemic pressure ratios, FFR: fractional flow reserve.

By the end of May 2023, NHPRs were measured simultaneously with FFR for 124 vessels in 221 patients during preoperative evaluations for open heart surgery. The vessels measured with NHPRs represented 47% of the vessels measured with FFR. Among 140 treated vessels, 66 vessels (47%) underwent NHPR measurement, while among 126 deferred vessels, 60 vessels (47%) also underwent NHPR measurement. The NHPRs measured were the iFR (14%), dPR (42%), DFR (9%), and RFR (35%) (Figure 4), according to the cardiologist’s preference.

### 3.3. Relation to FFR

Discordance between FFR and NHPRs is observed in up to 20% of cases [35]. Several factors are associated with FFR/NHPR discordance, including lesion location, age, multivessel disease, smoking, and hypertension. Lesions of the left main trunk, with a large perfusion area, may cause an underestimation by NHPRs. Lesions in the right coronary artery, with a lower diastolic-to-systolic flow velocity ratio, may also cause an underestimation by NHPRs [34]. Diffuse disease predominantly causes friction losses and abnormal NHPRs despite preserved FFR, while focal disease predominantly causes separation losses and abnormal FFR despite preserved NHPRs [35]. Therefore, we make the final decision to defer or treat coronary artery lesions based on FFR, although NHPRs are measured simultaneously.

### 3.4. NHPR Pullback

**Figure 5:**
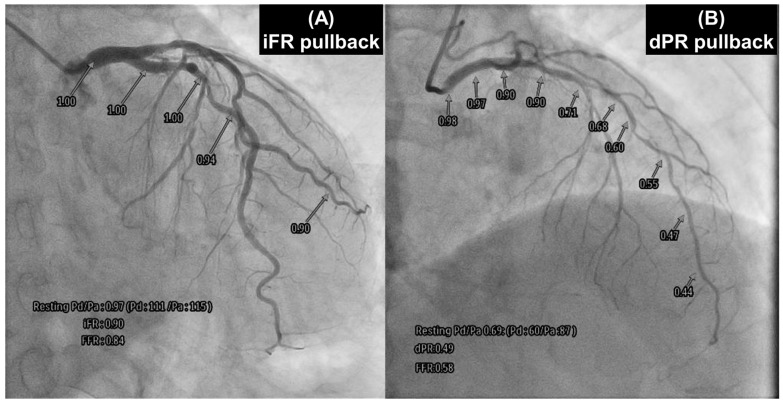
As NHPRs, iFR pullback (**A**) and dPR pullback (**B**) provide a trans-stenotic pressure gradient for each lesion in cases of tandem lesions. (**A**) A 75-year-old male patient, who was scheduled for aortic valve replacement due to aortic valve stenosis, showed 75% stenosis in the left circumflex artery on coronary angiography. To determine the need for revascularization, he underwent both FFR and pullback iFR measurements. The iFR values were >0.89 at all points, and the FFR was 0.84. Therefore, the lesion was deferred, and revascularization was not indicated. (**B**) A 73-year-old male patient presented with 75% stenosis in the left anterior descending (LAD) artery. He underwent both FFR and pullback dPR measurements. The dPR values gradually decreased (<0.89) as they were measured distally. The FFR was 0.58. Therefore, revascularization of the LAD was performed. NHPRs: non-hyperemic pressure ratios, iFR: instantaneous wave-free ratio, dPR: diastolic pressure ratio, LAD: left anterior descending artery.

In addition to the absence of a need for maximal hyperemia, NHPRs are suitable for longitudinal vessel measurements, with less crosstalk between serial stenoses compared to FFR [36]. NHPR pullback, as shown in Figure 5, may offer a potential solution by providing a trans-stenotic pressure gradient (ΔP) for each lesion in tandem lesions, especially in cases of FFR/NHPR discordance. Treating the lesion with the greatest ΔP first and then reevaluating the other lesion is a reasonable approach. It has been demonstrated that iFR pullback predicted post-PCI physiological values in tandem and diffuse lesions with an error of 1.4% [37]. It has also been shown that pre-PCI NHPR pullback predicted actual post-PCI NHPRs with high reliability [36]. Therefore, the number and total length of treated lesions may be lower with the NHPR pullback-guided strategy compared to the angiography-guided strategy. However, there is a paucity of data regarding NHPR pullback in CABG strategies. Serial lesion assessment using NHPR pullback is helpful in understanding the anatomy of coronary lesions with a greater pressure gradient. Long segments of diffusely diseased arteries should be more appropriately revascularized by CABG. Furthermore, the most suitable site for graft anastomosis can be identified based on NHPR pullback data.

## 4. Coronary CT Angiography and FFR-CT

**Figure 6:**
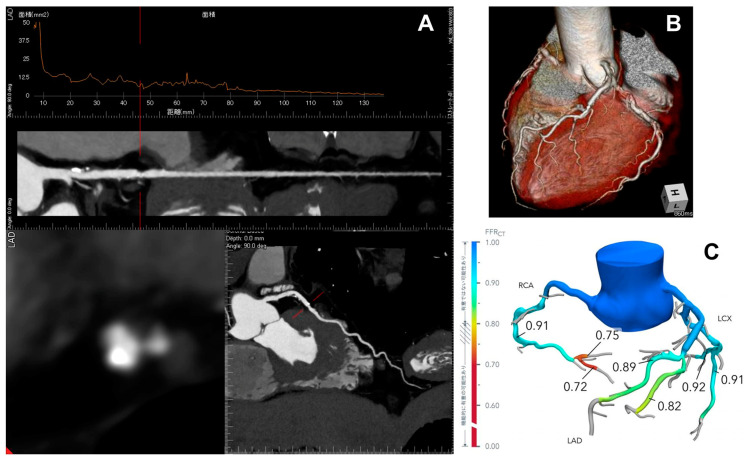
Quantitative computed tomographic coronary angiography (CTCA) (**A**), three-dimensional CTCA image (**B**), and fractional flow reserve (FFR-CT) measurement (**C**) in a 78-year-old male patient before surgery for a thoracic aortic aneurysm show moderate coronary lesions (50–70% lumen narrowing by visual CTCA) with partly calcified plaques in the right coronary artery (RCA) and left anterior descending artery (LAD). The RCA showed an FFR-CT value of 0.72 and was therefore grafted, whereas the LAD showed an FFR-CT value of 0.89 and was deferred.

Coronary tomographic coronary angiography (CTCA) has emerged as a non-invasive imaging modality for diagnosing coronary artery disease (CAD) with the advancement of new CT technologies. A recent systematic review and meta-analysis revealed that CTCA has diagnostic accuracy similar to direct invasive coronary angiography (ICA) and offers benefits compared with exercise ECG and SPECT-MPI [38]. Although lesions with a diameter stenosis ≥50% on visual CTCA are typically considered for referral to ICA, CTCA can provide quantitative information on coronary stenosis, lesion length, and plaque burden (Figure 6) [39].

The CTCA-derived anatomical parameters include lesion length, minimum lumen area (MLA), and % area stenosis. For example, an MLA cutoff value of 1.8 mm^2^ derived from CTCA has been reported as an anatomic predictor for physiological lesion significance [40]. However, this is smaller compared with the IVUS-derived cutoff. Additionally, it has been demonstrated that the anatomical assessment by CTCA correlates poorly with the functional significance of the stenosis defined by FFR [40]. The modest association between anatomical assessment by quantitative CTCA and functional assessment may be explained by the complexity of factors leading to myocardial ischemia.

FFR-CT has been introduced as a non-invasive method based on fluid dynamics algorithms developed by HeartFlow (HeartFlow Inc., Redwood, CA, USA), which simulate the effect of adenosine injection into the arteries and reproduce the invasive measurement based solely on the coronary anatomy provided by traditional CTCA (Figure 6) [41]. The PLATFORM trial confirmed the equivalent clinical outcomes of using CTCA plus FFR-CT compared to standard ICA in patients with stable, new-onset chest pain, highlighting the cost-effectiveness of FFR-CT and the reduction of unnecessary ICA procedures.

CTCA provides additional information for diagnosing CAD. One such feature is “intra-plaque dye penetration”, which refers to a contrast pool within the plaque but not contiguous with the lumen, a phenotype indicative of ruptured plaques [42]. Another feature is a lipid-rich necrotic core with intraluminal plaque hemorrhage, microcalcifications, or pre-existing plaque rupture, known as the “napkin-ring sign”, which increases the risk for future coronary events [43]. The ‘low-attenuation plaque’, defined by a fixed HU threshold of <30 HU, also correlates with the lipid-rich necrotic core of high-risk atherosclerotic plaques [44]. Furthermore, automated quantification of coronary plaque is feasible with CTCA using dedicated quantitative software [45]. Artificial intelligence (AI)-augmented CTCA allows for rapid, accurate evaluation of CAD and plaque characteristics, offering potential time and financial benefits along with enhanced reliability and accuracy in analysis [46].

These CTCA features are highly beneficial in PCI, as they significantly reduce cardiovascular mortality and the severity of angina compared to optimal medical therapy, while also improving the quality of life for patients with chronic coronary syndromes [47,48], particularly those with coronary chronic total occlusions (CTOs).

CTOs remain one of the most complex lesions in PCI, presenting significant technical challenges. Unfavorable histopathological features of CTOs, such as severe calcification, prevent wire advancement through the intraplaque space of the occlusion, resulting in higher periprocedural risk and potentially poorer outcomes. In cases of heavily calcified CTOs, CTCA provides invaluable non-invasive diagnostic information. For procedural planning in PCI, CTCA offers a comprehensive anatomical description of the coronary arteries, detailing both the length and course of the CTO lesions, as well as the extent of calcification throughout the occlusion. Consequently, higher procedural success rates and fewer complications have been reported for patients randomized to CTCA-guided CTO PCI compared to those who did not receive CTCA guidance [49].

Despite these advantages of CTCA in heavily calcified CTO cases, inherent limitations may reduce image quality. The resolution of the CT scanner used can impact the definition of critical features of the CTO lesion. Additionally, rendering and volume artifacts on CT imaging, often referred to as the “blooming effect”, can result in an erroneous enlargement of the calcified segment.

## 5. Quantitative Flow Ratio (QFR)

QFR is a novel computational approach that estimates FFR in real-time by using three-dimensional coronary artery reconstruction and computational fluid dynamics from standard angiograms. Previous studies have demonstrated a strong correlation between QFR and pressure wire-based FFR measurements [50,51]. In a large-scale, sham-controlled, blinded, randomized trial, lesion selection for PCI using QFR guidance improved clinical outcomes at 1 year by reducing procedural complications and improving long-term results compared to standard angiography-guided PCI [52]. These results were robust in both the intention-to-treat and per-protocol populations and were consistent across numerous pre-specified subgroups.

## 6. Future Perspectives

Cardiac surgeons have become familiar with FFR and NHPR measurements to evaluate myocardial ischemia. For them, non-invasive CTCA is highly anticipated, as it can provide simultaneous functional evaluation (myocardial ischemia) through FFR-CT and anatomical information via serial quantitative assessment. Dedicated software is being developed for the integration of CTCA in the catheterization laboratory to simplify CTCA-guided PCI [53]. Beyond the diagnostic phase, CTCA can also guide therapeutic interventions in a manner similar to structural heart interventions by adding 3D imaging to conventional angiography and incorporating the visualization of atherosclerotic plaques throughout the coronary tree. In the future, CTCA-guided CABG, which involves projecting CTCA images onto the surgical field in the operating room, may become a valuable strategy. In order to utilize CTCA image information for CABG, future challenges include collaboration with CT radiologists and automatic quantification of the images using AI.

## Figures and Tables

**Table 1:** Coronary artery evaluated by FFR before open heart surgery.

	Treated Vessels N = 140 (53%)	Deferred Vessels N = 126 (47%)	*p* Value
Patients	123 (57%)	94 (43%)	0.005
FFR	0.70 ± 0.08	0.87 ± 0.05	<0.001
Coronary lesion			
LAD	98 (70%)	56 (44%)	<0.001
Diagonal branch	2 (1%)	5 (4%)	
LCX	15 (11%)	35 (28 %)	
RCA	25 (18%)	30 (24%)	
Main surgery			
CABG	67 (55%)	19 (15%)	<0.001
Valve	43 (36%)	51 (40%)	
Aorta	11 (9%)	54 (43%)	
Others	0	2 (2%)	

FFR: fractional flow reserve, LAD: left anterior descending artery, LCX: left circumflex artery, RCA: right coronary artery, CABG: coronary artery bypass grafting.

## Data Availability

The raw data supporting the conclusions of this article will be made available by the authors upon request.
